# One-Pot Synthesis of SnO_2_-rGO Nanocomposite for Enhanced Photocatalytic and Anticancer Activity

**DOI:** 10.3390/polym14102036

**Published:** 2022-05-16

**Authors:** ZabnAllah M. Alaizeri, Hisham A. Alhadlaq, Saad Aldawood, Mohd Javed Akhtar, Maqusood Ahamed

**Affiliations:** Department of Physics and Astronomy, College of Sciences, King Saud University, Riyadh 11451, Saudi Arabia; zalaizeri@ksu.edu.sa (Z.M.A.); hhadlaq@ksu.edu.sa (H.A.A.); sdawood@ksu.edu.sa (S.A.); mjakhtar@ksu.edu.sa (M.J.A.)

**Keywords:** SnO_2_, rGO, nanocomposites, one-pot synthesis, characterization, photocatalytic degradation, anticancer activity

## Abstract

Metal oxide and graphene derivative-based nanocomposites (NCs) are attractive to the fields of environmental remediation, optics, and cancer therapy owing to their remarkable physicochemical characteristics. There is limited information on the environmental and biomedical applications of tin oxide-reduced graphene oxide nanocomposites (SnO_2_-rGO NCs). The goal of this work was to explore the photocatalytic activity and anticancer efficacy of SnO_2_-rGO NCs. Pure SnO_2_ NPs and SnO_2_-rGO NCs were prepared using the one-pot hydrothermal method. X-ray diffraction (XRD), transmission electron microscopy (TEM), scanning electron microscopy (SEM), X-ray photoelectron spectroscopy (XPS), Fourier transform infrared (FTIR), UV–Vis spectrometry, photoluminescence (PL), and Raman scattering microscopy were applied to characterize the synthesized samples. The crystallite size of the SnO_2_ NPs slightly increased after rGO doping. TEM and SEM images show that the SnO_2_ NPs were tightly anchored onto the rGO sheets. The XPS and EDX data confirmed the chemical state and elemental composition of the SnO_2_-rGO NCs. Optical data suggest that the bandgap energy of the SnO_2_-rGO NCs was slightly lower than for the pure SnO_2_ NPs. In comparison to pure SnO_2_ NPs, the intensity of the PL spectra of the SnO_2_-rGO NCs was lower, indicating the decrement of the recombination rate of the surfaces charges (e^−^/h^+^) after rGO doping. Hence, the degradation efficiency of methylene blue (MB) dye by SnO_2_-rGO NCs (93%) was almost 2-fold higher than for pure SnO_2_ NPs (54%). The anticancer efficacy of SnO_2_-rGO NCs was also almost 1.5-fold higher against human liver cancer (HepG2) and human lung cancer (A549) cells compared to the SnO_2_ NPs. This study suggests a unique method to improve the photocatalytic activity and anticancer efficacy of SnO_2_ NPs by fusion with graphene derivatives.

## 1. Introduction

Hepatocellular carcinoma is the second leading cause of death after lung cancer, globally [1]. The World Health Organization (WHO) reported that there were approximately 19 million new cancer cases and approximately 10 million deaths from cancer worldwide in 2020 [2]. There are several ways, such as surgery, radiation, and chemotherapy, to treat cancer. However, drug resistance and selectivity are still two major hurdles in cancer therapy. On the other hand, the release of environmental pollutants (e.g., dyes and drugs) from various factories challenge the intactness of the ecosystem. Hence, it is important to develop a nanostructure that can be used in biomedical as well as environmental applications [3].

Metal nanoparticles, such as silver nanoparticles (AgNPs), have recently become attractive to different areas such as catalysis, bio-sensing, antimicrobial activity, and bio-medicine due to the fact of their potential applications [4,5]. Metal oxide nanoparticles (NPs) have shown potential for their application in cancer therapy and environmental remediation [6,7]. For example, NPs of ZnO, TiO_2_, WO_3_, and In_2_O_3_ have been commonly studied for catalytic, anticancer, antibacterial, optoelectronics, and photocatalytic degradation [8,9,10] due to the fact of their unique physicochemical properties. Particularly, tin oxide (SnO_2_) NPs, an n-type semiconductor with a wide bandgap of ~3.6 eV, was studied for these purposes because of their several advantages, e.g., low cost, stability, facile synthesis, and low toxicity [11]. The hydrothermal method has been commonly used to synthesize various nanoforms of SnO_2_ such as NPs, nanorods, and nanowires [12,13]. Earlier studies reported the photocatalytic activity of SnO_2_ NPs under UV or visible light illumination [14,15]. However, there is a large scope for further improvement of the photocatalytic performance of such nanostructures.

Graphene is a 2D nanocrystal consisting of single layer of carbon atoms arranged in a honeycomb lattice. Graphene oxide (GO) and reduced graphene oxide (rGO) are the two most important derivatives of graphene [16,17]. GO and rGO are promising materials for various applications due to the fact of their interesting properties such as high surface area, high mechanical strength, excellent optical property, and presence of functional groups on their surfaces. Due to the presence of functional groups, GO and rGO have attracted the attention of material scientists for the synthesis of nanocomposites with metal oxide NPs [18,19]. Earlier studies focused on improving the physicochemical properties of metal oxide NPs by integration of GO or rGO [20,21]. Moreover, GO has different advantages that enhance the properties natural polymer as a nanocomposite. For example, natural polymer/GO composites (KGM/GO) are strong and biocompatible [22]. Different synthesis procedures, such as laser irradiation, gas–liquid interface interaction, co-precipitation, and hydrothermal, were employed to prepare metal oxide NPs anchored on GO/rGO nanosheets [23]. Such nanocomposites have superior characteristics than their individual one. For instance, ZnO-rGO NCs have better anticancer efficiency than pure ZnO NPs [20]. Au-rGO NCs were prepared by green synthesis for PTT of MCF7 cancer cell treatment with promising application in the field of nanobiomedicine [24]. Another study demonstrated that the photocatalytic activity of Fe3O4 NPs improved after doping with rGO [25]. Chen et al. [26] reported that the prepared Pd nanocatalyst exhibited an outstanding catalytic applicability for reducing extremely poisonous Cr(VI) and a broad variety of azo dyes.

Keeping the above in mind, we aimed to improve the anticancer and photocatalytic activity of SnO_2_ NPs by incorporating rGO. Pure SnO_2_ NPs and SnO_2_-rGO NCs were produced using the one-pot hydrothermal method. X-ray diffraction (XRD), field emission scanning electron microscopy (FE-SEM), field emission transmission electron microscopy (FE-TEM), X-ray photoelectron microscopy (XPS), Raman scattering microscopy, Fourier transmission infrared (FTIR) microscopy, UV–Vis spectrometry, and photoluminescence (PL) spectrometry were applied to characterize the physicochemical properties of the prepared samples. The photocatalytic activity of SnO_2_ NPs and SnO_2_-rGO NCs was examined against methylene blue (MB) dye under UV illumination. The anticancer potential of these samples were explored in two different cancer cells: human liver cancer (HepG2) and human lung cancer (A549).

## 2. Experimental Section

### 2.1. Materials and Reagents

Tine chloride (SnCl_4_·4H_2_O), graphite powder, sodium nitrate (NaNO_3_), sulphuric acid (H_2_SO_4_), potassium permanganate (KMnO_4_), hydrogen peroxide (H_2_O_2_), hydrazine monohydrate (NH_2_NH_2_·H_2_O), and methyl blue (MB) dye were obtained from Sigma Aldrich (Millipore-Sigma, St. Louis, MO, USA). Distilled water (DW) was employed as a medium for preparation. The chemicals were utilized as received without any further purification.

### 2.2. Synthesis of Graphene Oxide and Reduced Graphene Oxide

Graphene oxide (GO) was synthesized via Hummer’s method [27]. A reduction method was used to synthesize rGO to form GO. The 200 mg of GO was dispersed in 30 mL of DW. Then, 0.5 mL of hydrazine monohydrate (NH_2_NH_2_·H_2_O) was added to the above solution. The mixture solution was further warmed to 80 °C for 3 h to reduce GO into rGO. Subsequently, the suspension was centrifuged and rinsed several times with ethanol and distilled water and finally dried at 70 °C for 18 h to obtain the rGO sheets.

### 2.3. Synthesis of SnO_2_-rGO Nanocomposites

**Scheme 1:**
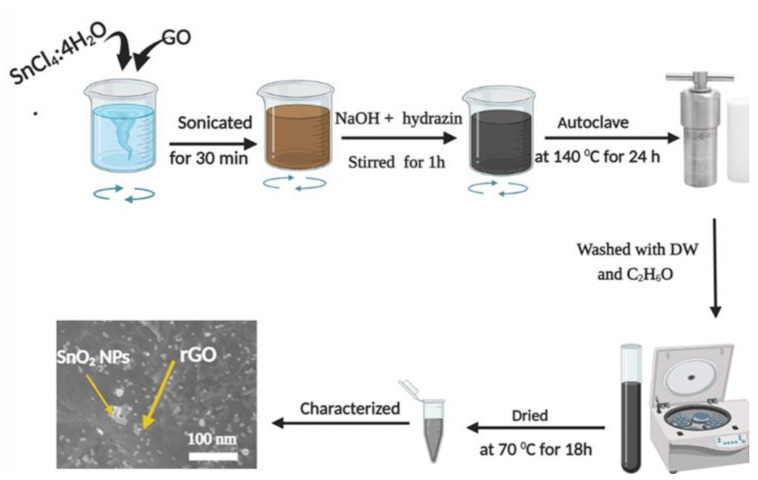
Synthesis protocol of the SnO_2_-rGO NCs.

A one-pot hydrothermal approach was applied to prepare the SnO_2_-rGO NCs. Briefly, 1 g of tine chloride (SnCl_4_·4H_2_O) and 0.2 g of GO were dispersed in 30 mL of DW and sonicated for 30 min. Then, 1.5 mL of hydrazine (NH_2_NH_2_·H_2_O) was added to this solution. The NaOH solution was further slowly added to the mixture to reach pH 13. Next, the mixture was stirred for 1 h to obtain a homogenous solution. Fifty milliliters was transferred to a stainless autoclave and heated at 140 °C for 24 h. After, the product was centrifuged, washed several times with ethanol and DW, and dried at 70 °C for 18 h to obtain the SnO_2_-rGO NCs. A similar procedure was applied for the synthesis of pure SnO_2_ NPs without mixing of GO. The synthesis protocol of the SnO_2_-rGO NCs is depicted in Scheme 1.

### 2.4. Characterization Techniques

X-ray diffraction (PanAnalytic XPert Pro, Malvern Instruments, Malvern, Worcestershire, UK), with Cu-Kα radiation (λ = 0.15405 nm, at 45 kV and 40 mA) and an angle ranging from 30° to 80°, was used to investigate the crystal structure and phase purity of the prepared samples. The morphology was studied via field emission transmission electron microscopy (FETEM) (200 kV, 2100F, JEOL, Inc., Tokyo, Japan). Mapping of the elemental distribution of the SnO_2_-rGO NCs was carried out by field emission scanning electron microscopy (FE-SEM) (JSM-7600F, JEOL, Inc.). Moreover, the chemical state and elemental composition of SnO_2_-rGO NCs were determined by X-ray photoelectron spectroscopy (XPS) (PHI-5300 ESCA PerkinElmer, Boston, MA, USA) and energy-dispersive X-ray spectroscopy (EDX) [28]. Moreover, the typical working conditions of XPS were 10^−9^ Torr. This is necessary because the released photoelectrons have a low energy and are easily absorbed by the surrounding environment. Al Kα with Mgα X-rays were used to excite the samples. A micro-Raman spectroscopic analysis was also performed using a Raman microscope (Thermo Scientific, Waltham, MA, USA) with a 532 nm (6 mW) laser and 32 two-second scans (IY-Horiba-T64000). The absorption spectra of the SnO_2_ NPs and SnO_2_-rGO NCs were recorded using a UV–Visible spectrophotometer (Hitachi U-2600). The PL peak intensity of SnO_2_ NPs and SnO_2_-rGO NCs were assessed by a fluorescent spectrometer (Hitachi F-4600, Hitachi, Tokyo, Japan) with an excitation wavelength of 300 nm. The effect of the rGO sheets on the microstructure of the SnO_2_ NPs was studied by Fourier transform infrared (FTIR) (PerkinElmer Paragon 500, Waltham, MA, USA).

### 2.5. Cell Culture

Human liver cancer (HepG2) and human lung cancer (A549) cell lines were used to assess the anticancer activity of NPs and NCs. Cell lines were purchased from American Type Culture Collection (ATTC, Manassas, WV, USA). Cells were cultured in DMEM (Invitrogen, Carlsbad, CA, USA) with 10% fetal bovine serum (FBS) and antibiotics (100 µg/mL streptomycin + 100 U/mL penicillin). Cells were maintained in a humidified incubator at 37 °C with a 5% CO_2_ supply.

### 2.6. Exposure Protocol

An amount of 1000 µg/mL of both of SnO_2_ NPs and SnO_2_-rGO NCs were dissolved in DMEM as stock suspension. The suspension was further diluted to different concentrations (i.e., 0, 5, 10, 25, 50, 100, and 200 µg/mL). The different dilutions NPs and NCs were sonicated for 20 min at 80 W at room temperature in an ultrasonic water bath sonicator before being exposed to cells.

### 2.7. Cell Viability

To evaluate the effect of the synthesized samples against two types of cancer (i.e., HepG2 and A549), MTT assays were performed. At a density of 1 × 10^4^ cells/well, 100 µL of cells were placed onto 96-well plates and incubated for 24 h. Then, 100 µL of each concentration (i.e., 0, 5, 10, 25, 50, 100, and 200 µg/mL) of the SnO_2_ NPs and the SnO_2_-rGO NCs were added into each well and incubated for 24 h. After, 100 µL of MTT solution was added to each well and incubated for 3 h. Next, 100 µL of CHAPS detergent (3-(3-cholamidopropyl) (dimethylammonio)-1-propanesulfonate) solution was added into each well to solubilize the MTT dye. Cell viability was then measured using a microplate reader (BioTek ELx800 Universal) at a wavelength of 570 nm.

### 2.8. Photocatalytic Evaluation

The photocatalytic experiments were conducted through degradation of MB dye under UV illumination (λ ≥ 420 nm), which was provided using a 400 W Xe lamp (CEL-HXF300, Beijing China Education Au-light Co., Ltd., Beijing, China). The 10 ppm of MB dye was dispersed in 50 mL of DI water under continuous stirring, and 2 mL of MB solution was taken as dye without catalysts. Then, 20 mg of both of SnO_2_ NPs and SnO_2_-rGO NCs was added to the above solution as a suspension solution. Before exposure to UV irradiation, an adsorption–desorption equilibrium was achieved after 30 min of stirring the suspension solution in the dark. At regular intervals, 2 mL of suspension under light was taken and centrifuged to remove the catalyst powders. The degradation efficiency of MB dye was estimated by following Equation (1).

(1) $$\mathrm{Degradation}\;\mathrm{efficiency}\;\left(\%\right)=\left[\frac{C_{0}-C_{t}}{C_{0}}\right]\times 100$$

where C_0_ represents the absorbance before irradiation, and C_t_ indicates the absorbance throughout the irradiation. To examine cycle stability, the catalysts were separated from suspension by centrifugation and subsequent washing with ethanol and deionized water. The catalysts were further dried at 60 °C for 12 h and utilized several times.

## 3. Results and Discussion

### 3.1. Crystallographic Study

**Figure 1:**
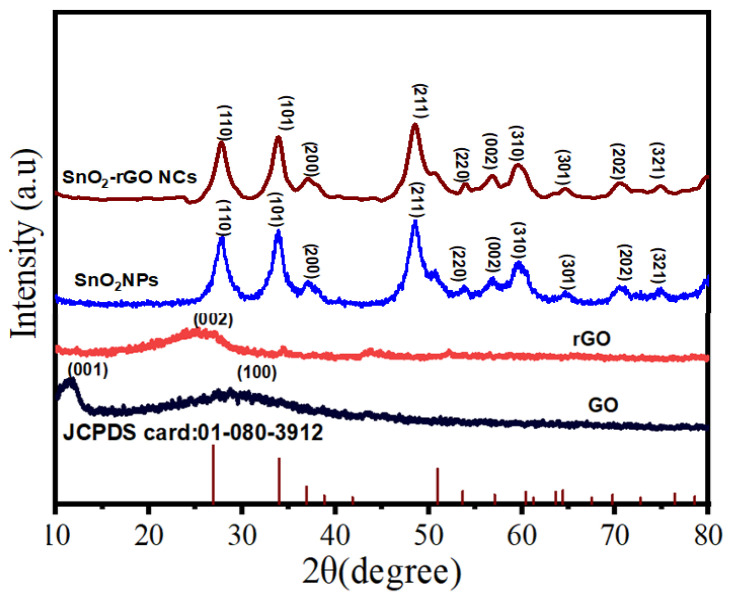
XRD spectra of GO, rGO, SnO_2_ NPs, and SnO_2_-rGO NCs.

The crystalline structure and phase formation of GO, rGO, SnO_2_ NPs, and SnO_2_-rGO NCs were examined by X-ray diffraction (XRD). The XRD spectra of GO and rGO nanosheets showed 2θ values of peaks at 10.57°, 27.94°, and 24.86° corresponding to the (001), (100), and (002) planes, respectively. In Figure 1, various 2θ (hkl) values of peaks are shown in the SnO_2_-rGO NCs at 26.24° (110), 33.92° (101), 37.94° (200), 51.60° (211), 58.30° (220), 61.8° (002), 78.48° (202), and 83.31° (321) planes. The results reveal that the peak of the (002) plane of graphitic carbon in the SnO_2_-rGO nanocomposites (NCs) overlapped with the (110) plane of the SnO_2_ NPs [29]. All of the SnO_2_ peaks in the XRD spectra associated to the standard SnO_2_ (JCPDS card No: 01-0803912). The average crystalline sizes of the SnO_2_ NPs and SnO_2_-rGO NCs were calculated using the Sherrier equation applying a (110) peak.

(2) $$D=\frac{\;k\lambda }{\beta \cos\theta }$$

where *k* = 0.90 is the shape factor of the crystallite, λ is the wavelength, β is the full width at half maximum, and θ is the reflection angle.

According to Scherrer’s equation, the average crystallite size of the SnO_2_ NPs and SnO_2_-rGO NCs were 7.64 and 7.98 nm, respectively (Table 1). The crystallite size of the SnO_2_ NPs decreased after loading on rGO as shown in other reports [30,31].

### 3.2. Morphological Study

**Figure 2:**
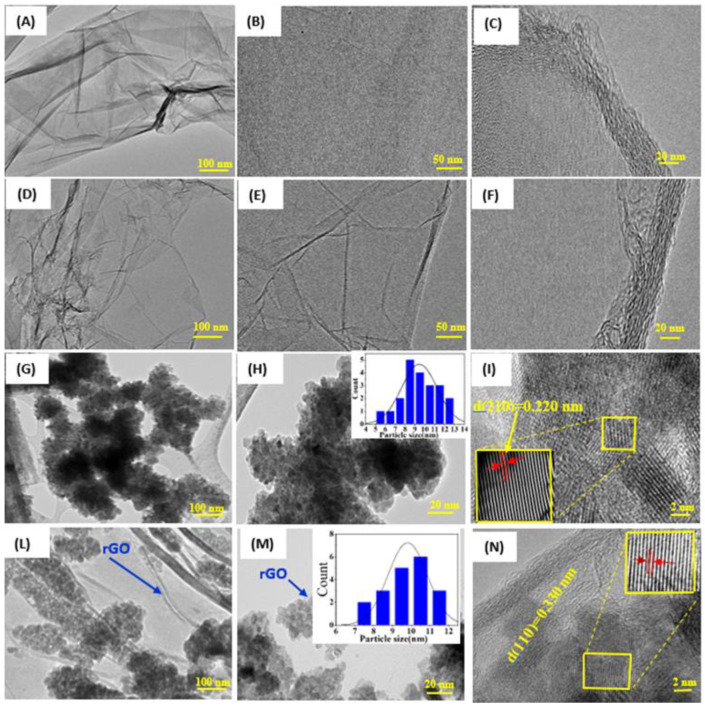
TEM characterization: TEM and HRTEM images of GO (**A**–**C**); rGO (**D**–**F**); SnO_2_ NPs (**G**–**I**); SnO_2_-rGO NCs (**L**–**N**).

High-resolution TEM (HRTEM) images were selected to investigate the morphology, chemical compositions, and particle size of the synthesized samples (Figure 2). TEM and HRTEM images show that GO exhibited thick, flattened nanosheet-like surfaces. Navazani et al. [32] reported that the increased thickness of the graphene sheets was attributed to organic functional groups and the electrostatic interaction of oxides on the surface. TEM images (Figure 2G,H) show that the distribution of the SnO_2_ NPs was randomly agglomerated with a uniform particles size owing to smaller particle size and higher surface energy of SnO_2_ NPs as reported earlier [33]. TEM images (Figure 2L,M) of the SnO_2_-rGO NCs show that the SnO_2_ NPs were loaded onto the rGO nanosheet. These results suggest the successful formation of SnO_2_-rGO NCs. Figure 2I,N show that the lattice spacing (d) of the SnO_2_ NPs and SnO_2_-rGO NCs were found to be 0.220 and 0.330 nm, respectively, which matched to the (210) and (110) planes of the SnO_2_ structure [34,35]. These values of interplanar spacing (d) were supported by the XRD data.

### 3.3. SEM Study

**Figure 3:**
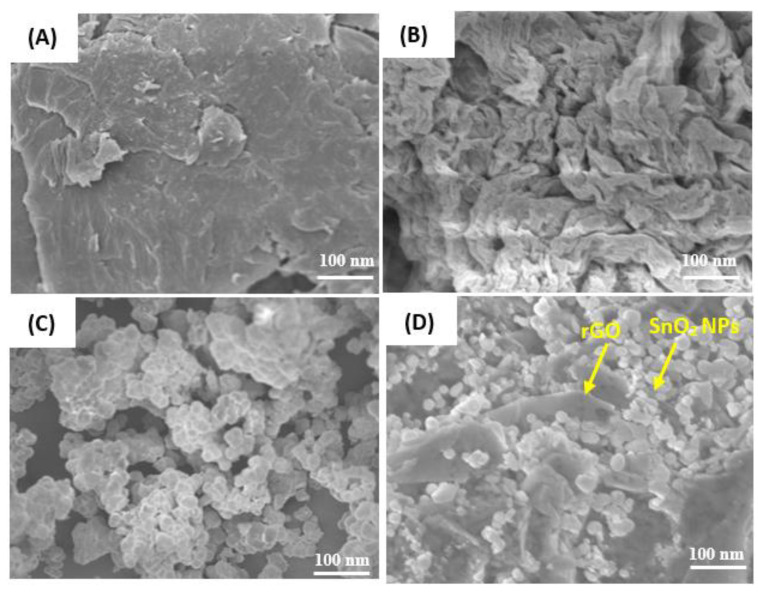
FE-SEM images of GO (**A**); rGO (**B**); SnO_2_ NPs (**C**); SnO_2_-rGO NCs (**D**).

**Figure 4:**
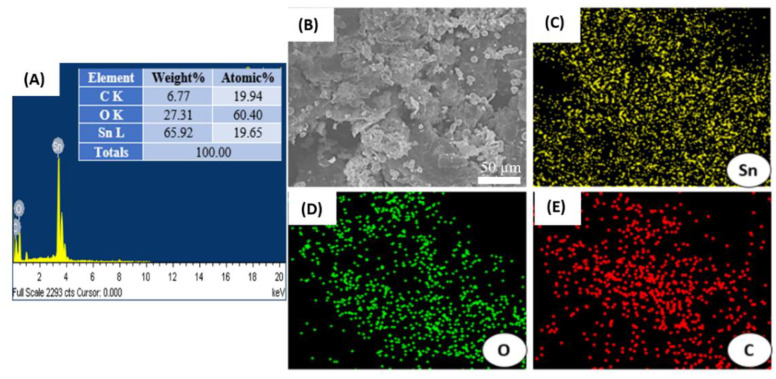
The elemental composition of the SnO_2_-rGO NCs by EDX (**A**). SEM elemental mapping of the SnO_2_-rGO NCs: (**B**) tin (Sn); (**C**) oxygen (O); (**D**); carbon (C) (**E**).

FE-SEM measurements were used to study the surface morphology of the prepared samples. Figure 3 displays the FE-SEM images of GO, rGO, SnO_2_ NPs, and SnO_2_-rGO NCs. In Figure 3A,B, the structure of GO and rGO nanosheets can be observed as a carbon layer. Figure 3C,D shows that the SnO_2_ NPs were spherical shaped with a uniform distribution and an anchored rGO sheet, which was also confirmed by TEM analysis. Ahamed et al. [36] observed that rGO improved the anticancer therapy of metal oxide NPs by tuning its physicochemical properties. Such a phenomenon is crucial and useful for photocatalytic degradation and cancer therapy. EDX spectra (Figure 4A) confirmed the presence of Sn, C, and O elements in the SnO_2_-rGO NCs. SEM mapping (Figure 4C,E) of SnO_2_-rGO NCs also exhibited a homogeneous distribution of Sn, O, and C elements in the SnO_2_-rGO NCs.

### 3.4. XPS Study

**Figure 5:**
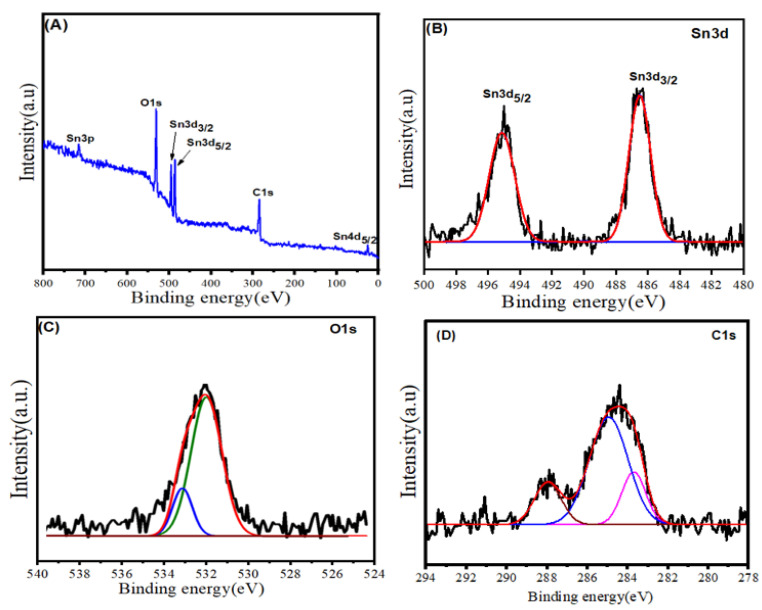
XPS wide spectra of SnO_2_-rGO NCs (**A**); Sn3d high-resolution XPS spectra (**B**); O1s high-resolution XPS spectra (**C**); C1s high-resolution XPS spectra (**D**).

The electronic structure and the chemical composition of the SnO2-rGO NCs samples were also investigated by X-ray photoelectron spectroscopy (XPS), thus providing information on the electronic communication between SnO2 and rGO [37]. The binding energy peaks of Sn, O, and C elements were shown in the XPS wide spectra (Figure 5A) of the SnO_2_-rGO NCs. It can be seen that peaks of the Sn element were matched to the spin-orbit peaks of Sn 3p, Sn 3d, and Sn 4p [38]. The high-resolution spectra of the Sn, O, and C are presented in Figure 5B–D, respectively. The results suggest that the presence of Sn, O, and C peaks confirm the fabrication of SnO_2_ on rGO as supported by the EDX data (Figure 4A). The binding energy of the Sn3d_5/2_ and Sn3d_3/2_ peaks were assigned at 486.55 and 495.3 eV, respectively, which could be attributed to the SnO_2_ NPs [39]. According to the curve fit of the O1s spectra (Figure 5C), the binding energy peaks of O1s at 532 and 533 eV corresponded to Sn-O and Sn-O-C, respectively, which agrees with previous works [40,41]. The C1s peak (Figure 5D) was subdivided into three individual peaks after the fitting of the main peak. The peaks at 284, 285, and 288 eV were observed to correspond to C=C, C-OH, and O-C=O, respectively, as shown in a previous study [42]. These results reveal that the rGO had a residual oxygen group [40].

### 3.5. Raman Analysis

**Figure 6:**
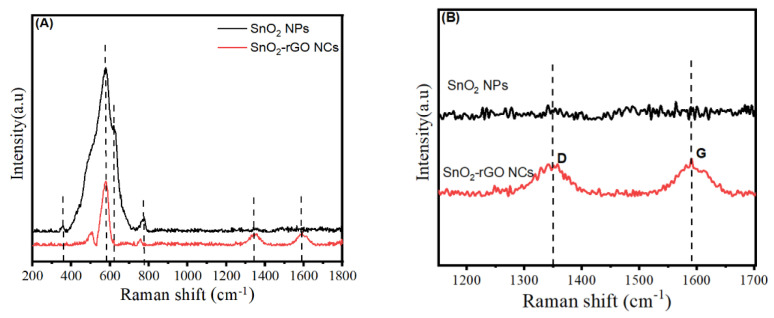
Raman spectra of the SnO_2_ NPs and SnO_2_-rGO NCs (**A**); magnified Raman spectra of the SnO_2_ NPs and SnO_2_-rGO NCs (**B**).

The electronic and vibration bands of prepared samples were analyzed via Raman spectroscopy. The Ramen spectra of the SnO_2_ NPs and SnO_2_-rGO NCs are shown in Figure 6A. It can be seen that E_u_, A_s_, A_1g_, and B_2g_ vibration bands of the SnO_2_ NPs’ peaks were located at 352.33, 579, 620, and 773.32 cm^−1^, respectively [43]. The peak positions at 579 and 620 cm^−1^ of the SnO_2_-rGO NCs were similar to the SnO_2_ NPs as shown in Figure 6A. These results indicate that the intensity of the Raman peaks of the SnO_2_-rGO NCs was lower than the SnO_2_ NPs due to the interaction between SnO_2_ NPs and rGO [39]. Figure 6B shows that two new scattering bands (i.e., band D and band G) appeared in the SnO_2_-rGO NCs compared to the SnO_2_ NPs. Moreover, the scattering bands of the SnO_2_-rGO NCs were observed at 1349.47 cm^−1^ (band D) and 1590.50 cm^−1^ (band G), which were attributed to instability in the hexagonal graphitic layers and vibration bonds of the carbon atom, respectively [44]. Our results from the Raman analysis confirm that the SnO_2_ NPs attached onto the surface of the rGO sheets [45].

### 3.6. FTIR Study

**Figure 7:**
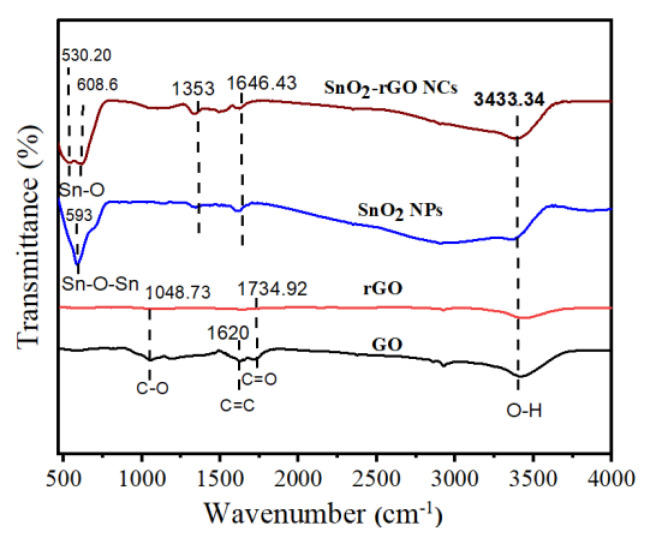
FTIR spectra of the prepared GO, rGO, SnO_2_ NPs, and SnO_2_-rGO NCs.

The functional groups of the GO, rGO, SnO_2_ NPs, and SnO_2_-rGO NCs were determined by FTIR spectrometry. Figure 7 shows the FTIR spectra of the prepared samples in a range of 450–4000 cm^−1^. The results reveal the changes in the microstructural characteristics in the first region (500–650 cm^−1^) of the SnO_2_ NPs during loading on rGO. However, three stretching vibrations of C-O, C=C, and C=O assigned at 1048.73, 1620, and 1734.92 cm^−1^, respectively, can be seen in the GO. The presence of C=O and C-O at the 1713.96 and 1048.37 cm^−1^ bands indicate the reduction of GO into rGO [46]. Moreover, O-H stretching vibrations were observed at 3433.69, 1646.43, and 1353 cm^−1^ for both SnO_2_ NPs and SnO_2_-rGO NCs due to the existence water molecules during the hydrothermal process [47]. The band observed at 593 cm^−1^ was assigned to the O-Sn-O stretching mode in the SnO_2_ NPs [48], while two strong peaks at 530.20 and 608.6 cm^−1^ in the SnO2-rGO NCs were assigned to Sn-O stretching vibration owing to the strong interaction between SnO2 and rGO [49]. These results indicate the formation of single-structure SnO_2_-rGO NCs as supported by the XRD data.

### 3.7. Optical Study

**Figure 8:**
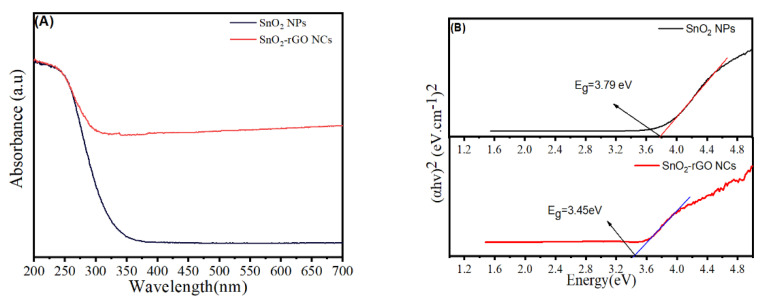
UV–Vis spectra of the SnO_2_NPs and SnO_2_-rGO NCs (**A**); the bandgap energy of the SnO_2_ NPs and SnO_2_-rGO NCs (**B**).

The optical properties of the SnO_2_ NPs and SnO_2_-rGO NCs were studied by UV–Vis spectroscopy. The UV–Vis spectra of the SnO_2_ NPs and SnO_2_-rGO NCs are presented in Figure 8A. The absorption edge was shifted toward a higher wavelength for the SnO_2_-rGO NCs in comparison to pure SnO_2_ NPs. The difference in absorption peaks tuned the bandgap energy of the SnO_2_-rGO NCs in comparison to the SnO_2_ NPs. Taucs formula was used to assess the bandgap energy [50]. The bandgap energy (E_g_) (Figure 8B) of the SnO_2_ NPs and SnO_2_-rGO NCs were 3.79 and 3.45 eV, respectively. We observed a slight decrease in the bandgap energy of the SnO_2_ NPs after rGO integration due to the increase in particle size as shown Table 1. Reduction of the bandgap energy improves UV light absorption, which can be applied in the photocatalytic degradation of pollutants [51].

### 3.8. Photoluminescence (PL)

**Figure 9:**
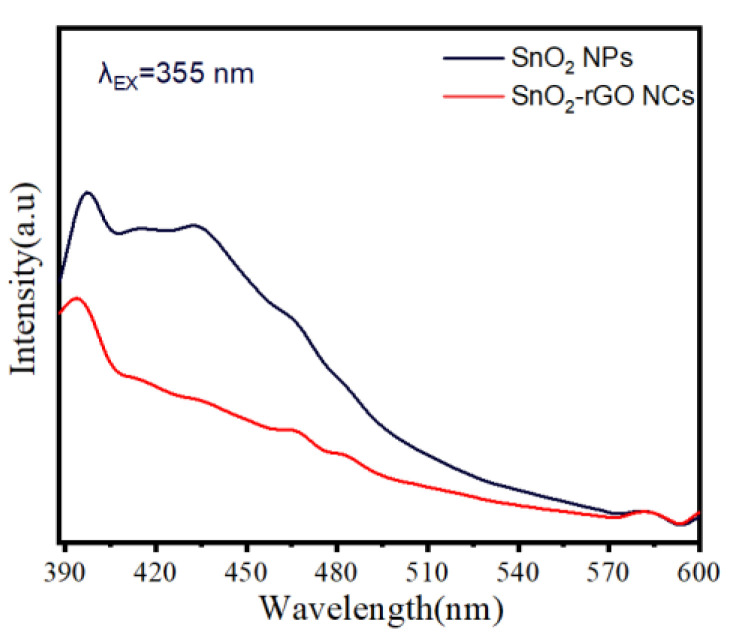
PL spectra of the SnO_2_NPs and SnO_2_-rGO NCs.

Photoluminescence (PL) was used to measure the migration rate of electrons and holes in the SnO_2_NPs and SnO_2_-rGO NCs NPs. The PL spectra of the SnO_2_NPs and SnO_2_-rGO NCs NPs are presented in Figure 9. Moreover, the PL spectra of the SnO_2_ NPs and SnO_2_-rGO NCs at room temperature were obtained using an excitation wavelength of 355 nm. After adding rGO, an emission peak at 402 nm was greatly reduced. Our results observed that the PL intensity of the SnO2-GO NCs system (black line) was lower than that of pure SnO2 NPs, which may be attributed to greater charge separation, a longer life of the electron–hole pair, and a higher efficiency of charge [52]. This process has the potential to be used in a large variety of applications including cancer treatment and the photocatalytic degradation of environmental contaminants.

### 3.9. Photocatalytic Study

**Figure 10:**
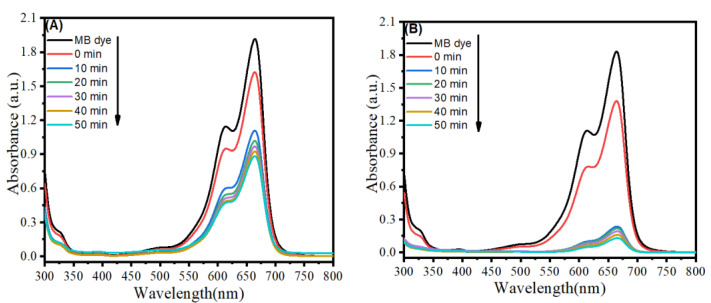
UV–Vis absorbance spectra for the degradation of the MB dye at different time intervals under UV irradiation: SnO_2_ NPs (**A**); SnO_2_-rGO NCs (**B**).

The degradation of MB dye under UV irradiation was used to investigate the photocatalytic activity of the SnO_2_ NPs and SnO_2_-rGO NCs. Figure 10A,B shows the absorption spectra of the MB solution of the SnO_2_ NPs and SnO_2_-rGO NCs under UV irradiation within 50 min. The absorption wavelength of the MB solution was at 664 nm as shown in Figure 10. We observed that the intensity of the absorption at 664 nm in the MB spectra decreased with increasing exposure time. Additionally, the color of the MB solution faded gradually, indicating the breaking of the dye’s structure by oxidation [53]. Ali et al. [54] also observed that MB dye was degraded by nanocomposites (NCs) under UV irradiation over a short exposure time.

**Figure 11:**
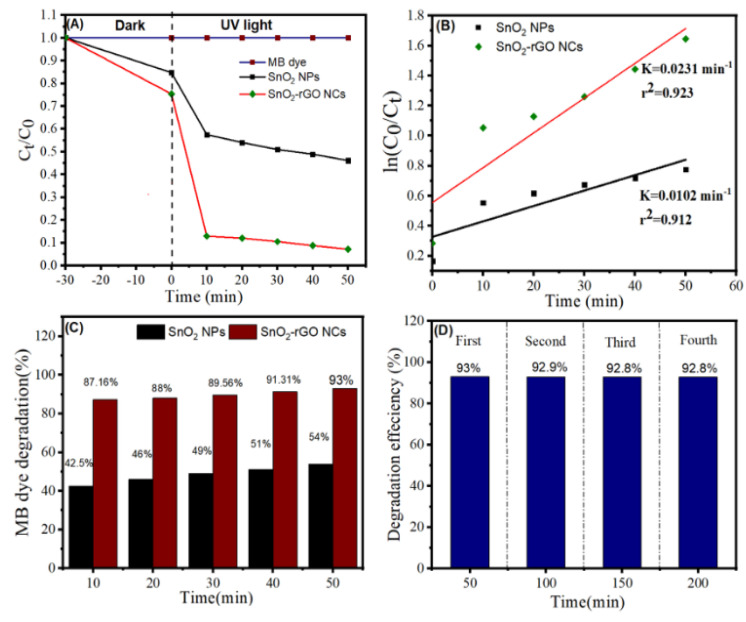
Variations in (C_t_/C_0_) as a function of irradiation time for SnO_2_ NPs and SnO_2_-rGO NCs (**A**), The plot of ln(C_0_/C_t_) as a function of light exposure time (**B**), the degradation efficiency of the SnO_2_ NPs and SnO_2_-rGO NCs (**C**), and four cycling runs of the SnO_2_-rGO NCs for the degradation of the MB dye under UV irradiation (**D**).

Figure 11A depicts the variations in (C_t_/C_0_) as a function of exposure time for pure SnO_2_ NPs and SnO_2_-rGO NCs at different time intervals (0–50 min) under UV irradiation. The photocatalytic activity of the nanocomposites was enhanced by rGO integration [55]. The results show that the degradation efficiency of the SnO_2_-rGO NCs (93%) under UV light after 50 min was higher than for the SnO_2_ NPs (54%). The reaction kinetics of the current catalysis can be described as pseudo-first-order kinetics, i.e., ln(Ct/C0) = −kt (k = kinetic rate constant, t = reaction time, C_0_ = initial absorbance, and C_t_ = absorbance at time t). The rate constant (K) values of the SnO_2_ NPs and SnO_2_-rGO NCs were 0.0231 and 0.0102 min^−1^, respectively (Figure 11B). Figure 11D displays the recyclability of the SnO_2_-rGO NCs after a four-time run with the same experimental conditions. The catalysts were washed in ethanol and deionized water and centrifuged to test for cycle stability. Then, catalysts were dried at 60 °C for 12 h and used multiple times. Even after four runs, the degradation efficiency of the MB dye was still approximately 93%. This indicates that the photocatalytic performance of the SnO_2_-rGO NCs can be continuously used without damage during the oxidation of contaminants. Our results indicate that the SnO_2_-rGO NCs had outstanding stability and potential for application in environmental remediation [56]. A comparison of the present samples’ degradation efficiency of MB dye with previous studies is shown in Table 2.

### 3.10. Mechanism of Photocatalysis

**Figure 12:**
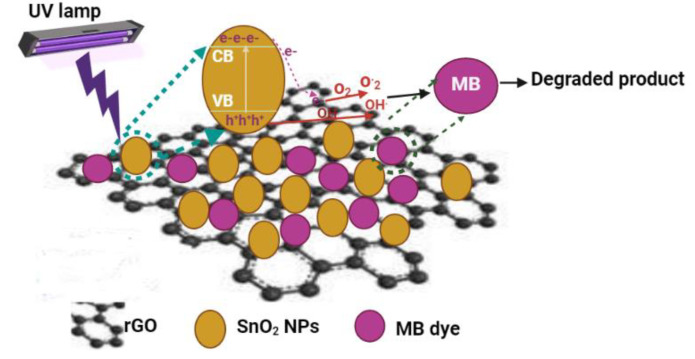
Photocatalytic mechanism for the degradation of MB dye by the SnO_2_-rGO NCs.

The photocatalytic mechanism for the degradation of MB dye by SnO_2_-rGO NCs is shown in Figure 12. The MB dye degradation process occurs via a series of chemical reactions, which generate superoxide and hydroxyl radicals. Prepared samples (SnO_2_ NPs and SnO_2_-rGO NCs) and MB dye were exposed under UV radiation (λ<400 nm). Upon irradiating the SnO_2_, excited electrons in the valance band (VB) transferred to the conduction band (CB), producing holes in the VB (Equation (3)). The electrons in the CB of the SnO_2_ NPs could transfer to the rGO due to the SnO_2_ NPs attached to the rGO surfaces (Equation (4)). The generated electrons (e^−^) and holes (h^+^) reacted with water (H_2_O) to produce new free oxygen radicals (Equation (5)). These radicals can be transferred to the hydroxyl radicals (Equations (6) and (7)). Hydroxyl radicals were produced by water molecules and holes (Equation (8)). These free oxygen radicals can decompose MB dye into smaller molecules (Equation (9)). The rGO reduced the rate of electron–hole pair recombination in SnO_2_-rGO NCs, which further increased the degradation efficiency of the SnO_2_-rGO NCs.

(3) $${\mathrm{SnO}}_{2}-\mathrm{rGO}\to {\mathrm{SnO}}_{2}-\mathrm{rGO}\left(e^{-}+h^{+}\right)$$

(4) $${\mathrm{SnO}}_{2}\left(e^{-}\right)\to e^{-}{}_{\mathrm{trap}}(\mathrm{rGO})$$

(5) $$e^{-}{}_{\mathrm{trap}}\left(\mathrm{rGO}\right)+O_{2}\to O_{2}^{•}$$

(6) $$O_{2}^{•}+2{\mathrm{HO}}^{•}+H^{+}\to H_{2}O_{2}+O_{2}$$

(7) $$H_{2}O_{2}\to 2{\mathrm{HO}}^{•}$$

(8) $$h^{+}{}_{\mathrm{VB}}+\mathrm{OH}/H_{2}O\to {\mathrm{HO}}^{•}+H^{+}$$

(9) $${\mathrm{HO}}^{•}+\mathrm{MB}\;\mathrm{dye}\to \mathrm{degraded}\;\mathrm{product}$$

### 3.11. Anticancer Study

**Figure 13:**
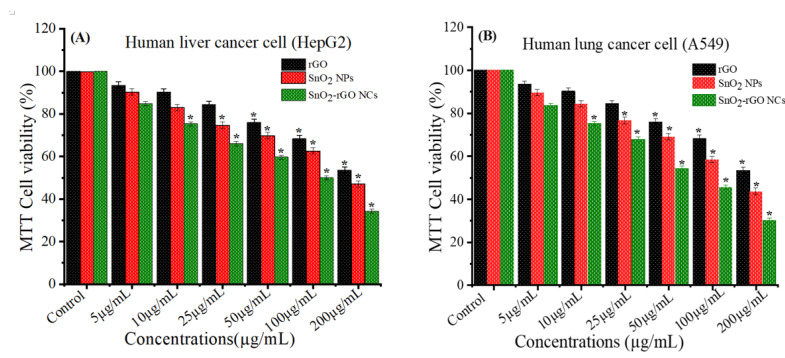
Cytotoxicity of rGO, SnO_2_ NPs, and SnO_2_-rGO NCs in human liver cancer HepG2 cells (**A**) and lung cancer A549 cells (**B**); one-way ANOVA followed by the post hoc test was applied for statistical analysis. * *p* < 0.05, statistically significant difference when compared to the control group.

Several recent studies showed that the anticancer potential of metal oxide NPs can be enhanced by integration with graphene derivatives [62,63]. For instance, Ahamed et al. [36] observed that SnO_2_-ZnO-rGO NCs displayed higher cytotoxicity toward human breast cancer cells (MCF-7) in comparison to pure ZnO NPs to improve their physiochemical properties such as the bandgap energy, which was lower compared to SnO_2_-ZnO NPs and pure ZnO NPs. In this work, we focused on improving the cytotoxicity of SnO_2_ NPs against cancer cells by rGO incorporation. The cytotoxicity at different concentrations (5-200 µg/mL) of SnO_2_ NPs and SnO_2_-rGO NCs was assessed in human liver cancer (HepG2) and lung cancer (A549) cells (Figure 13A,B). The results indicate that both SnO_2_ NPs and SnO_2_-rGO NCs induced dose-dependent cytotoxicity in the two types of cancer cells. Moreover, the SnO_2_-rGO NCs exerted a higher cytotoxicity than the pure SnO_2_ NPs, and the anticancer potential of the SnO_2_ NPs increased with rGO doping. The IC50 values of the SnO_2_-rGO NCs were almost 1.5-fold higher in both cancer cells than for pure SnO_2_ NPs (Table 3). These results indicate that rGO integration effectively enhanced the anticancer efficacy of the SnO_2_ NPs. In addition, these data warrant further research on the anticancer potential of SnO_2_-rGO NCs in suitable in vivo models.

## 4. Conclusions

In summary, SnO_2_ NPs and SnO_2_-rGO NCs were successfully prepared using a novel one-pot hydrothermal approach. The XRD data showed that the average crystallite size of the SnO_2_ NPs decreased after rGO doping. HRTEM and SEM results revealed that SnO_2_ NPs tightly anchored the rGO sheets, and SnO_2_ and rGO were uniformly distributed in the SnO_2_-rGO NCs with high-quality lattice fringes without distortion. The XPS and EDX data confirmed the chemical state and elemental composition of the SnO_2_-rGO NCs. Optical data suggested that the bandgap energy of the SnO_2_-rGO NCs was slightly lower than that of the pure SnO_2_ NPs. The intensity of the PL spectra was lower in the SnO_2_-rGO NCs in comparison to the pure SnO_2_ NPs. In the photocatalytic test, the MB degradation efficiency of the SnO_2_-rGO NCs (93%) was higher than for the SnO_2_ NPs (54%). This can be explained by a lower bandgap energy and lower PL intensity, which reduced the separation of charge carries (i.e., electrons and holes). Moreover, the anticancer efficacy of SnO_2_-rGO NCs was almost 1.5-fold higher against human liver cancer HepG2 and lung cancer A549 cells in comparison to pure SnO_2_ NPs. These data suggest a novel approach to enhance the photocatalytic activity and anticancer performance of SnO_2_ NPs via rGO fusion. This study warrants further research on SnO_2_-rGO NCs for the photocatalytic degradation of different pollutants and anticancer efficacy in various cancer and normal cells.

## Figures and Tables

**Table 1:** Structural and Optical Properties of the SnO_2_ NPs and SnO_2_-rGO NCs.

Properties	SnO_2_ NPs	SnO_2_-rGO NCs
XRD size (nm)	7.64	7.98
TEM size (nm)	9.42	9.84
Optical bandgap (eV)	3.79	3.45

**Table 2:** Comparison of the MB dye degradation efficiency of the present samples with previous studies.

Sample	Model Dye Pollutant	Reaction Time	Degradation Efficiency (%)	Concentration of MB	Source of Light	Reference
SnO_2_-rGO NCs	Methylene blue (MB)	50 min	93%	10 ppm	UV irradiation	This work
ZnO/GONCs	Methylene blue (MB)	40 min	100%	10 ppm	Sunlight irradiation	[57]
TiO_2_/rGO/Ag NCs	Methylene blue (MB)	120 min	91.2%	10 ppm	UV irradiation	[58]
rGO/ZnO/Cu	Methylene blue (MB)	60 min	95.14%	5 ppm	Sunlight irradiation	[59]
BiOBr/rGO NCs	Methylene blue (MB)	75 min	96%	7 ppm	Sunlight irradiation	[60]
Au/WO_2.72_/ rGONCs	Methylene blue (MB)	60 min	91.2%	10 ppm	Sunlight irradiation	[61]

**Table 3:** IC_50_ values of SnO_2_ NPs and SnO_2_-rGO NCs for two types of human cancer cells.

Samples	Human Liver Cancer (HepG2) Cells	Human Lung Cancer (A549) Cells
SnO_2_ NPs	160.97 μg/mL	153.13 μg/mL
SnO_2_-rGO NCs	100 μg/mL	95.72 μg/mL

## Data Availability

The data presented in this study are available on request from the corresponding author.
